# Knockdown of aquaporin-8 induces mitochondrial dysfunction in 3T3-L1 cells

**DOI:** 10.1016/j.bbrep.2015.09.009

**Published:** 2015-09-18

**Authors:** Reina Ikaga, Iyuki Namekata, Vassilios N. Kotiadis, Haruko Ogawa, Michael R. Duchen, Hikaru Tanaka, Naoko Iida-Tanaka

**Affiliations:** a Department of Advanced Biosciences, Graduate School of Humanities and Sciences, Ochanomizu University, 2-1-1 Otsuka, Bunkyo-ku, Tokyo 112-8610, Japan; b Department of Food Science, Otsuma Women's University, Chiyoda-ku, Tokyo 102-8357, Japan; c Department of Pharmacology, Toho University Faculty of Pharmaceutical Sciences, Funabashi, Chiba 274-8510, Japan; d Department of Cell and Developmental Biology, University College London, London WC1E 6BT, UK

**Keywords:** TMRE, Tetramethylrhodamine Ethyl ester, FCCP, Carbonyl cyanide 4-(triflouromethoxy) phenylhydrazone, Aquaporin-8, 3T3-L1 cells, Mitochondria, Adipocyte

## Abstract

**Background:**

Aquaporin-8 (AQP8), a member of the aquaporin water channel family, is expressed in various tissue and cells, including liver, testis, and pancreas. AQP8 appears to have functions on the plasma membrane and/or on the mitochondrial inner membrane. Mitochondrial AQP8 with permeability for water, H_2_O_2_ and NH_3_ has been expected to have important role in various cells, but its information is limited to a few tissues and cells including liver and kidney. In the present study, we found that AQP8 was expressed in the mitochondria in mouse adipose tissues and 3T3-L1 preadipocytes, and investigated its role by suppressing its gene expression.

**Methods:**

AQP8-knocked down (shAQP8) cells were established using a vector expressing short hairpin RNA. Cellular localization of AQP8 was examined by western blotting and immunocytochemistry. Mitochondrial function was assessed by measuring mitochondrial membrane potential, oxygen consumption and ATP level measurements.

**Results:**

In 3T3-L1 cells, AQP8 was expressed in the mitochondria. In shAQP8 cells, mRNA and protein levels of AQP8 were decreased by about 75%. The shAQP8 showed reduced activities of complex IV and ATP synthase; it is probable that the impaired mitochondrial water handling in shAQP8 caused suppression of the electron transport and ADP phosphorylation through inhibition of the two steps which yield water. The reduced activities of the last two steps of oxidative phosphorylation in shAQP8 cause low routine and maximum capacity of respiration and mitochondrial hyperpolarization.

**Conclusion:**

Mitochondrial AQP8 contributes to mitochondrial respiratory function probably through maintenance of water homeostasis.

**General significance:**

The AQP8-knocked down cells we established provides a model system for the studies on the relationships between water homeostasis and mitochondrial function.

## Highlights

• AQP8 was expressed in mouse adipose tissues and 3T3–L1 preadipocytes.
• AQP8 was localized in mitochondria in 3T3–L1 cells.
• Knockdown of AQP8 lowered routine and maximum capacity of mitochondrial respiration.
• Knockdown of AQP8 reduced activities of complex IV and ATP synthase in mitochondria.
• AQP8 contributes to maintain mitochondrial respirationprobably via water excretion.

## 1 Introduction

The major function of mitochondria is the production of ATP by oxidative phosphorylation. Through this process, a large amount of water is also formed. At the last step of the electron transport chain (ETC) mediated by cytochrome c oxidase (complex IV), electrons are finally passed to oxygen to yield water. In addition, ATP synthase-mediated production of ATP is coupled with equimolar formation of water. Thus, production of water as a byproduct of ATP synthesis is inevitable and appropriate water handling is of crucial importance for the maintenance of mitochondrial integrity and function. Concerning the water transport across the mitochondrial membrane, channel mediated pathways such as aquaporins and the mitochondrial permeabilization pore have been postulated in addition to simple lipid bilayer diffusion. However, the details of such facilitated pathways remain largely unknown and more information has to be obtained.

Aquaporins (AQPs) are a family of membrane transport proteins which play a role in water transport across biological membranes. At least thirteen AQPs (AQP0~12) have been identified in mammals. Most of them are localized in the plasma membrane and are involved in water transport into and/or out of cells and specific functions of the cells; reabsorption of water in proximal tubular cells, water permeability of the blood-brain-barrier, and salivary secretion [1], [2].

AQP8 was initially thought to be localized to the plasma membrane when it was cloned and identified in testis, liver, colon and heart [3], [4], [5], [6]. But later it was found to be present on the inner mitochondrial membrane of liver, kidney and neural stem cells and was expected to play a role in unconditional transport of metabolic water produced in mitochondria [7]. AQP8 was recently reported to be involved in the mitochondrial transport of H_2_O_2_ in the hepatocytes [8] and NH_3_ in the renal tubular cells [9] and hepatocytes [10]. However, the fact that AQP8 also shows high permeability to water comparable or superior to that of AQP1 [11] suggests its involvement in mitochondrial water flux. To achieve a comprehensive understanding of AQP8 function, accumulation of experimental evidence in various types of cells is needed.

The mitochondrial type AQP8 was distinct from the plasma membrane form in terms of molecular weight. In many cell types, AQP8 is simultaneously expressed in the plasma membrane and mitochondria. In the liver and testis, the two organs which show most active AQP8 synthesis, AQP8 was detected by western blot analyses as two bands; the band of about 28kDa was observed in the inner mitochondrial membrane-enriched fraction [12], [13] and the other one of about 34kDa in the plasma membrane fractions [12], [14], [15], [16], [17]. The 34kDa band was converted to the 28kDa band by *N*-glycosidase treatment [16], [17].

In this study, we found that, in mouse adipose tissues and 3T3-L1 preadipocytes, the mitochondrial type AQP8 is dominantly expressed. We therefore created an AQP8-knocked down cell line in which we have investigated the role of AQP8 on mitochondrial function, and showed that knockdown of AQP8 leads to significantly impaired mitochondrial function, suggesting a significant role for AQP8 for normal mitochondrial bioenergetic homeostasis.

## 2 Methods and materials

### 2.1 Cell and cell culture

Mouse 3T3-L1 preadipocytes obtained from Health Science Research Resources Bank (Osaka, Japan) were cultured in Dulbecco's modified Eagle's medium high glucose (DMEM, GIBCO) supplemented with 100 Unit/ml penicillin, 100 µg/ml streptomycin, 20 mM HEPES and 10% calf serum at 37 °C under 5% CO_2_ atmosphere.

### 2.2 Animals

Male BALB/cCrSlc and male C57BL/6J mouse at 8 weeks and 10 weeks of age were obtained from Charles River Laboratories International, Inc. and Sankyo Labo Service Corporation, Inc., respectively.

### 2.3 Preparation of mitochondria and mitoplast fractions

Cells washed with PBS were resuspended in mitochondrial isolation buffer (20 mM HEPES, 220 mM mannitol, 70 mM sucrose, 1 mM EDTA, 1 mM PMSF, *p*H 7.6), incubated on ice for 20 min and homogenized using Biomasher (Nippi). Supernatant clarified at 800 g for 10 min was pelleted at 10,000 g for 20 min yielding a crude mitochondrial fraction. The pellet was resuspended in mitochondrial isolation buffer, clarified at 800 g for 20 min and pelleted at 10,000 g for 20 min resulting in an enriched mitochondrial fraction [18].

The preparation of mitoplast fraction was performed by using a detergent to remove the outer mitochondrial membrane. Digitonin was added to the mitochondrial fraction resuspended in mitochondrial isolation buffer to a final concentration of 0.6% (*w*/*v*). The suspension was incubated on ice for 15 min under gentle stirring. After dilution with 3 vol of isolation buffer, the suspension was centrifuged at 15,000 g for 10 min and the pellet was washed in mitochondrial isolation buffer resulting in an mitoplast fraction [8], [9].

### 2.4 Western blotting

Sample preparation: Cells, mitochondria or mitoplasts were lysed in cold RIPA buffer consisting of 25 mM Tris–HCl, pH 7.4, 150 mM NaCl and 1% NP-40 (Thermo Scientific), supplemented with a protease inhibitor cocktail (Sigma-Aldrich). Adipose tissues were homogenized in cold Lysis buffer (Cell Signaling), which consists of 20 mM Tris–HCl, pH 7.5, 150 mM NaCl, 1 mM Na_2_EDTA, 1 mM EGTA, 1% Triton, 2.5 mM sodium pyrophosphate, 1 mM β-glycerophosphate, 1 mM Na_3_Vo_4_ and 1 µg/ml leupeptin, supplemented with 1 mM PMSF. After centrifugation of the cell lysates or tissue homogenates at 14,000 g for 10 min at 4 °C, the protein concentration was measured using by DC^TM^ Protein Assay Reagent (Bio-Rad).

Immunoblotting: Lysates of whole cells, mitochondria or mitoplasts (5 to 25 µg protein) or tissue homogenates (25 or 60 µg protein) were loaded on a 10-20% gradient or 12.5% SDS-Page gel (ATTO), separated and transferred onto a PVDF membrane (Hybond-ECL, GE Healthcare). The membrane was incubated with 2% ECL-Advance blocking agent (Amersham Scientifices) in TBS containing 0.1% tween 20 (TBS-T) for 1.5 to 2 hours at room temperature. After washing with TBS-T, the membrane was incubated with a goat polyclonal antibody specific for AQP8 (Santa Cruz Biotechnology) or Total OXPHOS Rodent WB Antibody Cocktail (Abcam) at 4 °C overnight. After washing with TBS-T, it was incubated with HRP-conjugated anti-goat IgG (Santa Cruz Biotechnology) or anti-mouse IgG (Cell Signaling). The blots were developed by a Pierce Western Blotting Substrate Plus (Thermo scientific). The luminescence-active bands were observed using a Lumi Cube plus (Liponics) and the band intensities were quantified using Image J. For re-probing with anti-beta-actin antibody (Santa Cruz Biotechnology), the antibodies on the membranes were stripped by incubating with 2% SDS/100 mM 2-mercaptoethanol in 62.5 mM Tris–HCl buffer (pH6.8) for 30 min at 55 °C.

Enzyme degradation: Cell lysates containing 150 μg protein were incubated with 1 U of *N*-glycosidase F (Roche) in 20 mM sodium phosphate buffer (*p*H 7.5) containing 10 mM EDTA, 0.1% SDS, 0.2% 2-mercaptethanol and 1.0% IGEPAL-630 at 37 °C for 24 to 72 h followed by immunoblotting as described above.

### 2.5 Immunofluorescence staining

The cells were rinsed with PBS and fixed in cold acetone for 10 min at 4 °C. Subsequently, the cells were washed in PBS and incubated with PBS containing 0.5% Triton X-100 for 4 min at room temperature. After washing with PBS, the cells were incubated with 10% fetal bovine serum (FBS) in PBS for 60 min. After removing the buffer, the cells were incubated for 60 min at room temperature with 0.2% FBS / 3% BSA in PBS containing goat polyclonal antibody specific for AQP8 (Santa Cruz Biotechnology) together with rabbit polyclonal antibody specific for VDAC1 (Abcam) or mouse monoclonal antibody specific for cytochrome c (Cell Signaling). After three times washing in PBS containing 0.1% Tween-20 (PBS-T), the cells were incubated for 60 min at room temperature with 0.2% FBS / 3% BSA in PBS containing Alexa Fluor 546-conjugated anti-goat antibody (Invitrongen) together with Alexa Fluor 488-conjugated anti-rabbit antibody (Invitrogen) for VDAC1 staining or Alexa Fluor 488-conjugated anti-mouse antibody (Invitrogen) for cytochrome c staining. After three times washing with PBS-T and three washes in PBS, the fluorescence images were obtained by using confocal microscope (LSM700, Carl Zeiss) and analyzed by using LSM Image Browser (Carl Zeiss).

### 2.6 Establishing stable knockdown of AQP8

To obtain stably AQP8-knocked down cells, 3T3-L1 cells were transfected with a plasmid producing short hairpin RNA targeting AQP8. The plasmid was designed on pBAsi-mU6 Neo vector by TaKaRa and the target sequence was CTATCGGTCATTGAGAATA. As the negative control cells, 3T3-L1 cells were transfected with a plasmid including a nonsense sequence, TCTTAATCGCGTATAAGGC. Cells were seeded in 6-well plates two days before transfection. For each well containing the cells in about 60% confluency, 3 µg of the plasmid DNA in TransIT-LT1 Transfection reagent (TaKaRa) was added. Stably knocked down cells were selected by culturing in the presence of 3.5 mg/ml of G418, a neomycin analog (Geneticin, GIBCO) and maintained with 1.0 mg/ml of G418. For AQP8-over expressed cells, 3T3-L1 cells were transfected with a pcDNA3.1(+) vector carrying mouse AQP8 cDNA and the cells stably expressing AQP8 were selected as described above.

### 2.7 Quantitative real-time PCR

Preparation of total RNA from cells was performed with ISOGEN (NIPPON GENE). The cDNA was synthesized by using the Prime script RT reagent (TaKaRa). Real-time PCR was performed on Applied Biosystems 7300 Real Time PCR System by using the SYBR Green Master Mix (TaKaRa). Primers used for detection of AQP8 were 5ʹ-CGGATGTCTATCGGTCATTGAGAA-3ʹ and 5ʹ-GCGACACAGCAGGGTTGAAG-3ʹ and those for housekeeping gene beta-actin were 5ʹ-CATCCGTAAAGACCTCTATGCCAAC-3ʹ and 5ʹ- ATGGAGCCACCGATCCACA-3ʹ. Data were shown as the fold differences normalized to the β-actin.

### 2.8 Measurement of mitochondrial membrane potential

The mitochondrial membrane potential was evaluated using the potentiometric indicator tetramethylrhodamine ethyl ester, TMRE [19], [20]. TMRE fluorescence in the AQP8-knocked down cells and control cells was simultaneously observed in a single view and their fluorescence intensities were compared. For distinguishing cell lines, cell membrane specific fluoroprobe PKH67 (Sigma-Aldrich) was used. The AQP8-knocked down or control cells were labeled by PKH67, mixed with unlabeled counterparts and re-seeded on coverslips. The mixed cells were treated with the 25 nM TMRE probe for 30 min at room temperature in the recording chamber. The fluorescence images were obtained with a confocal microscope (LSM700, Carl Zeiss) and analyzed by using LSM Image Browser (Carl Zeiss).

### 2.9 Analyses of oxygen consumption and mitochondrial complex IV activity

The oxygen consumption rates of AQP8-knocked down cells and control cells were measured by high-resolution respirometry with the Oxygraph-2k (OROBOROS INSTRUMENTS) in recording medium (20 mM HEPES/10% FBS in DMEM). After measuring the baseline Routine respiration rate, we sequentially added to the recording medium, 2.5 µM oligomycin A, 2 µM FCCP and 2.5 µM antimycin A, to obtain the levels of Leak, maximum Uncoupled, and residual non mitochondrial oxygen consumption, respectively. To measure mitochondrial complex IV activity, the cells were treated with 2 mM Ascorbate and 0.5 mM TMPD (N, N, Nʹ, Nʹ-Tetramethyl-p-phenylenediamine dihydrochloride) in the presence of antimycin A. The software DataLab (OROBOROS INSTRUMENTS) was used for data acquisition and analysis.

### 2.10 Analysis of cellular ATP level

Intracellular ATP levels were measured with the CellTiter-Glo Luminescent Cell Viability Assay (Promega). Counted cells were seeded onto opaque-walled 96-well plates and were lysed in the same plate with the reagent included in the assay kit for 10 min at 37 °C. Luminescence produced from ATP-mediated chemical reaction was read by a luminometer (Thermo scientific).

### 2.11 Measurement of mitochondrial complex I activity

Mitochondrial complex-I activity was measured by determining the decrease in NADH absorbance at 340 nm that leads to the reduction of ubiquinone (CoQ1) to ubiquinol as described by Ragan et al. with slight modification [21], [22]. The assay was initiated by the addition of 50 µM CoQ1 to the reaction mixture containing homogenized cells (9×10^4^ cells), 20 mM of potassium phosphate buffer (pH 7.2), 10 mM MgCl_2_, 2.5 mg/ml BSA and 1 mM KCN. After monitoring the reaction for 5 min, 20 µM rotenone was added, the solution was left for 3 min to let the rotenone take effect and the reaction was monitored continuously for a further 5 min. The activity was calculated using the extinction co-efficient of 6.81 mM^−1^ cm^−1^ for NADH.

### 2.12 Cell viability assay

Trypan blue exclusion assay: After culturing for 24 h, cells attached to culture dishes were trypsinized and collected by centrifugation. Floating cells in the medium were also collected by centrifugation and the cells were combined all together. The collected cells were resuspended in the medium and were combined with equal volumes of 0.4% (*w*/*v*) trypan blue dye solution (GIBCO) and analyzed microscopically on a Neubauer counting chamber. Blue cells were counted as nonviable, and cells excluding the dye were counted as viable. Cell viability was expressed as percentage of viable cells [8].

Lactate dehydrogenase (LDH) assay: LDH activity in the culture medium was measured to assess cell viability using LDH assay kit (Dojindo Labo). At 1.5 h after culturing in medium without serum, released activity in medium and total activity after cell lysis were sequentially measured. The percentage of LDH release was determined by dividing the LDH released into the medium by the total LDH (released plus cellular).

### 2.13 Electron microscopy

Cells were fixed with 2% paraformaldehyde/2% glutaraldehyde in 0.1 M phosphate buffer (pH 7.4) at 37 °C and kept for 30 min at 4 °C. They were then fixed with 2% glutaraldehyde in 0.1 M phosphate buffer (pH 7.4) at 4 °C overnight, after which samples were analyzed by Tokai Em, Inc [23]. Samples were rinsed three times with 0.1 M phosphate buffer (pH 7.4) for 30 min each, followed by postfixation with 2% osmium tetroxide (OsO_4_) in 0.1 M phosphate buffer (pH 7.4) at 4 °C for 1 h. Fixed samples were dehydrated, embedded in Quetol-812 (Nisshin EM), and polymerized at 60 °C for 48 h. The blocks were ultrathin-sectioned at 70 nm with a diamond knife using a Leica ultramicrotome. Sections were placed on copper grids and stained with 2% uranyl acetate at room temperature for 15 min and then rinsed with distilled water, followed by secondary staining with lead stain solution (Sigma-Aldrich) at room temperature for 3 min. The grids were observed under a JEOL JEM-1400Plus transmission electron microscope at an acceleration voltage of 80 kV.

### 2.14 Statistical analyses

The data were expressed as mean±S.D. The significance of the difference between the mean values of groups was evaluated by Student's *t* test. Results were considered significant if either *p<0.05 or **p<0.01

## 3 Results

### 3.1 AQP8 is expressed in 3T3-L1 cells and mouse adipose tissues, and localizes to mitochondria

**Fig. 1:**
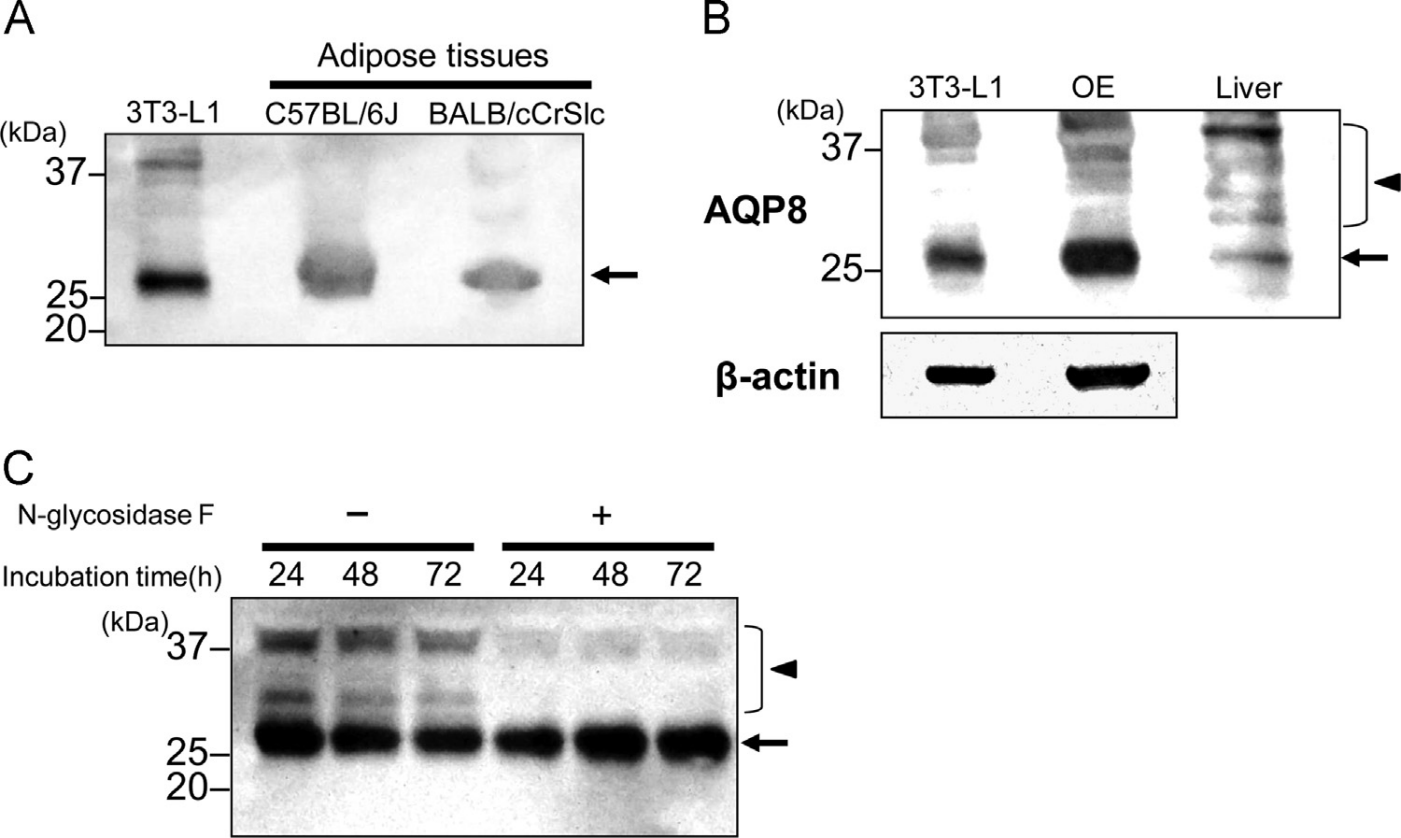
Expression of AQP8. A: Western blotting of AQP8 in 3T3–L1 cell lysate and homogenates of adipose tissue in C57BL/6J and BALB/cCrSlc mice. The lane for 3T3–L1 cells was loaded with 5 µg of protein and those for adipose tissues with 60 µg. The molecular weight of the band pointed by the arrow was estimated to be approximately 28kDa. B: Western blots for the expression of AQP8 in lysates of 3T3–L1 cells and AQP8-overexpressed cells (OE) and tissue homogenates of liver in C57BL/6J mice. The amount of sample applied was 5 µg protein for the cell lysates and 25 µg protein for the liver. C: *N*-glycosidase treatment of AQP8 proteins in 3T3–L1 cell lysate. Aliquots (25 µg protein) of the cell lysate were digested with (+) and without (-) *N*-glycosidase F for 24, 48, or 72 h at 37 °C. The bands pointed by the arrow-head, which have molecular weights of 30–38 kDa, were markedly reduced after the digestion.

In western blots (Fig. 1A), an AQP8-immunoreactive band of 28kDa (arrow), corresponding to the molecular weight of the mitochondrial type AQP8, was detected as the major one in 3T3-L1 cells and adipose tissues of C57BL/6J and BALB/cCrSlc mice. Additionally a minor band of about 38kDa was observed in 3T3–L1 cells. Fig. 1B shows that the 38kDa band was the major one in mouse liver homogenate and that a few minor bands in 30–38 kDa range were observed in addition to the 28kDa band in the liver. The bands in 30–38kDa were observed in the AQP8-overexpressed 3T3–L1 cells (Fig. 1B). They were also expressed in wild type of 3T3–L1 cells as extremely minor bands and were markedly reduced by *N*-glycosidase digestion (Fig. 1C), suggesting that all of them are glycosylated type AQP8s and that multiple species of glycosylated type AQP8 exist in mouse liver and 3T3–L1 cells. These results are consistent with previous reports indicating that liver expressed both types of AQP8, that is, non-glycosylated and glycosylated types. On the other hand, 3T3–L1 cells and adipose tissues predominantly express the non-glycosylated type which has been considered to be expressed in mitochondria.

**Fig. 2:**
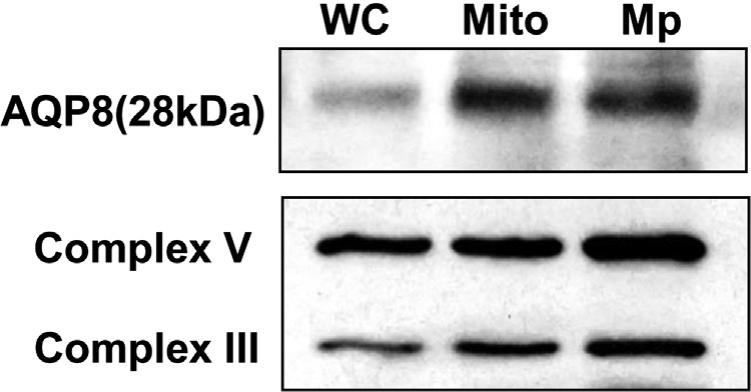
Expression of AQP8 in mitochondria and mitoplast fractions in 3T3–L1 cells. Western blotting of whole cell (WC), mitochondria (Mito) and mitoplast (Mp) lysates prepared from 3T3–L1 cells. Each lane was loaded with 6 µg protein. Complex III and V were used as markers of the inner mitochondrial membrane.

**Fig.3:**
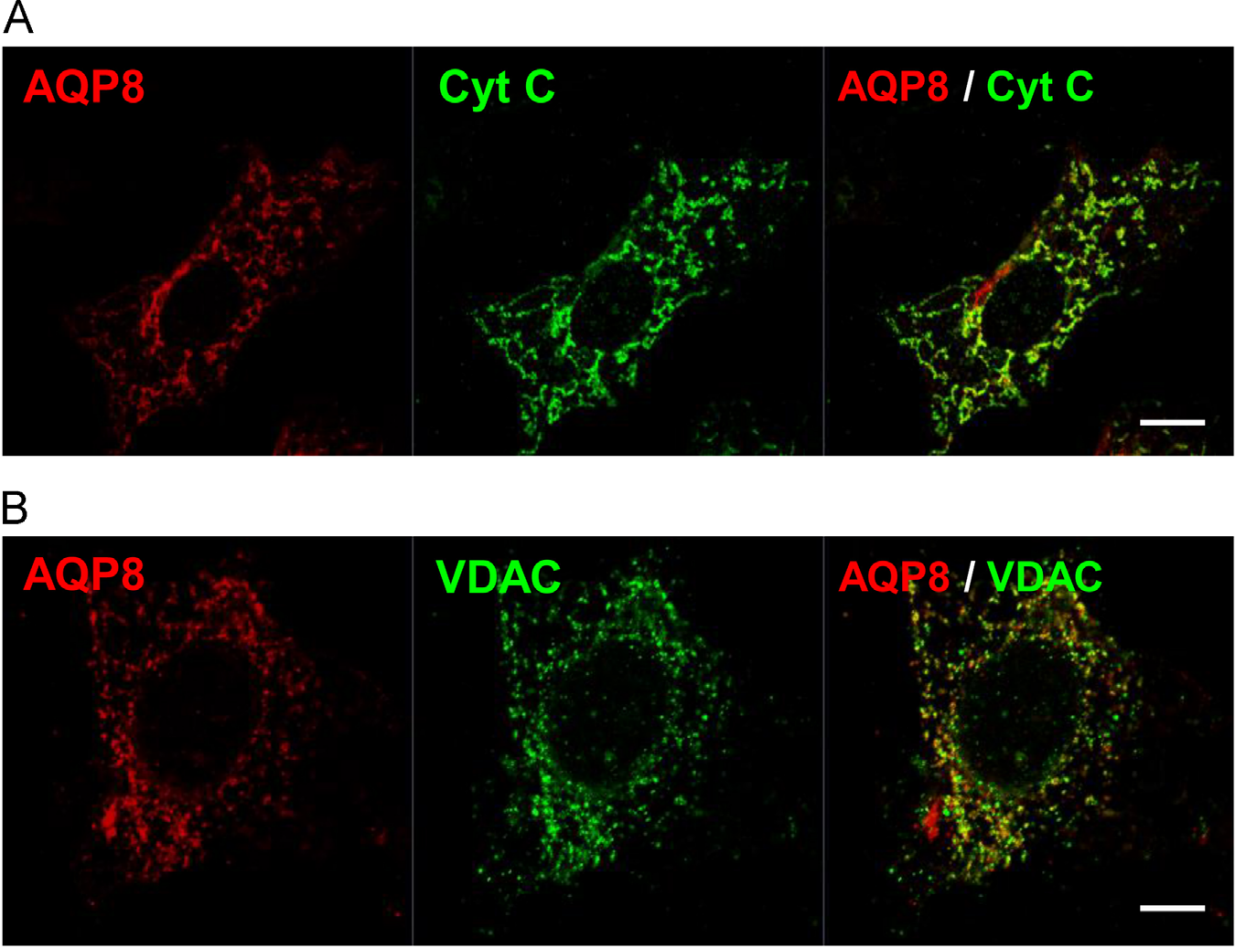
Localization of AQP8 in 3T3–L1 cells. AQP8 in 3T3–L1 cells was double-immunostained with mitochondrial marker proteins, cytochrome c (A: Cyt C) and voltage-dependent anion channel (B: VDAC) and were observed by confocal fluorescence microscopy. Scale bar, 10 µm. Colocalization analyses were performed with four independent experiments for both Cyt C and VDAC.

To confirm the mitochondrial localization of AQP8 in 3T3–L1 cells, western blotting of purified mitochondria and mitoplast (Fig. 2) and immunofluorescence staining (Fig. 3) were performed. In western blotting, the 28kDa mitochondria type band was more intense in the mitochondria fraction and the mitoplast than in the whole cell lysate. The same intensity pattern was observed with the inner mitochondria membrane marker Complex III and V. Fig. 3 shows co-localization of AQP8 with mitochondrial marker proteins, cytochrome c and the voltage-dependent anion channel (VDAC). Especially, the AQP8 fluorescence coincided well with cytochrome c fluorescence. The percentages of colocalized pixels with cytochrome c or VDAC to all pixels of AQP8 were 90.6±2.8 and 85.1±5.8%, respectively. These results confirm that AQP8 is localized to the mitochondria in 3T3–L1 cells.

### 3.2 Establishing stably AQP8-knocked down cells

**Fig. 4:**
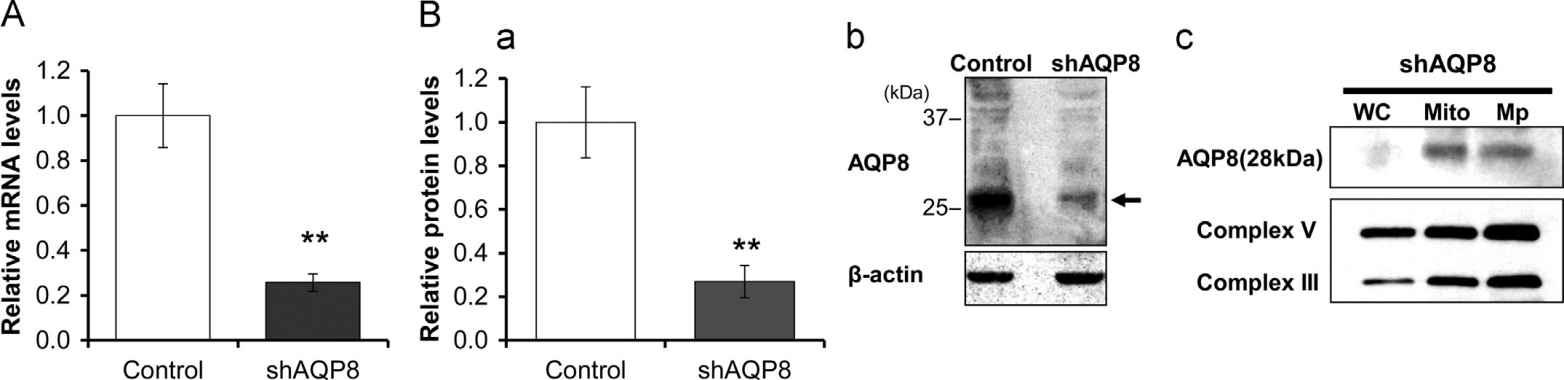
Levels of the AQP8 expression in AQP8-knocked down cells. A: Summarized results for the AQP8 mRNA levels in 3T3–L1 cells assessed by real-time PCR. AQP8 mRNA levels were normalized to those of β-actin, and the mRNA levels of shAQP8 cells were expressed as a ratio against the controls. Data are expressed as the mean±S.D. of three independent experiments. B: Summarized results (a) and a typical western blot image (b) for the protein levels of AQP8 in shAQP8 cells. Each lane was loaded with 15 µg of protein. Intensities of the band pointed by the arrow, which has molecular weight of approximately 28kDa, were normalized to those of β-actin and expressed as the protein levels of AQP8. Data are expressed as the mean±S.D. of five independent experiments. (**p<0.01). (c) Western blots of whole cell (WC), mitochondria (Mito) and mitoplast (Mp) prepared from shAQP8 cells. Each lane was loaded with 6 µg of protein.

To investigate the function of AQP8 in the mitochondria, we transfected the 3T3–L1 cells with a plasmid coding shRNA designed against AQP8, and a clone with high ability of differentiation to adipocytes was selected and named shAQP8. The mRNA level of AQP8 in the shAQP8 cells was less than 26±4% of control cells (Fig. 4A). Western blot analysis showed that the amount of the 28kDa protein in shAQP8 cells was about 27±7% of that in the control cells (Fig. 4B(a) and (b)). In shAQP8 cells, the band of 28kDa was the major one and its intensity was increased in mitochondria and mitoplast fractions as observed in 3T3–L1 cells, suggesting that the localization of AQP8 is the same as 3T3–L1 cells (Fig. 4B(c)).

### 3.3 Mitochondrial membrane potential in AQP8-knocked down cells

**Fig. 5:**
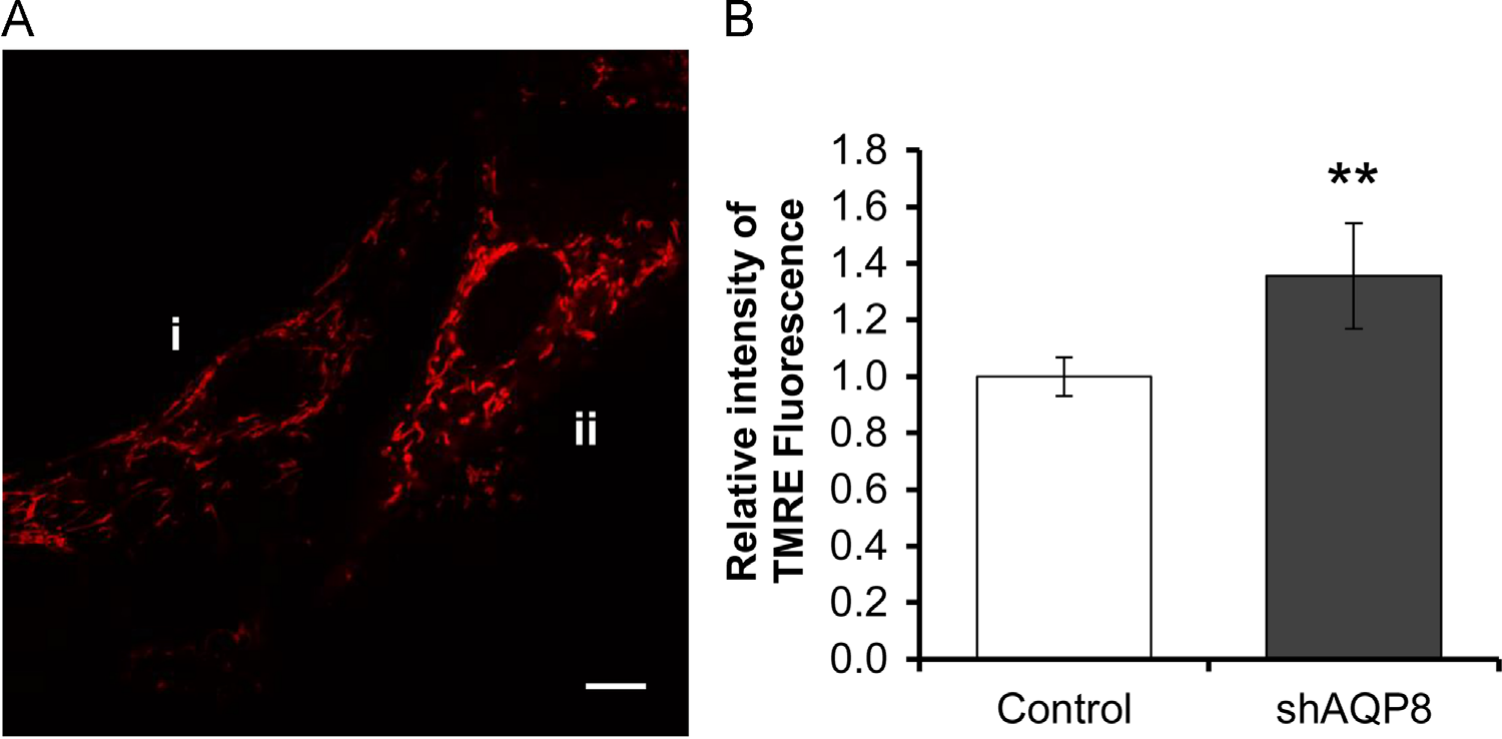
Levels of mitochondrial membrane potential. A: Typical fluorescence images of TMRE in control cells (i) and in an shAQP8 cell (ii). The control cells in the image shown were labeled by PKH67. B: Summarized results for the TMRE fluorescence. The fluorescence intensities in shAQP8 cells were normalized against the controls. Data were expressed as the mean±S.D. of total twelve independent experiments (**p<0.01), in which the control cells were labeled in the six experiments and the shAQP8 cells were labeled in another six. Scale bar, 10 µm.

The mitochondrial membrane potential was evaluated by using fluorescence microscopy and TMRE. The TMRE fluorescence intensity of the shAQP8 cells was significantly (1.36±0.19-fold) higher than the control cells (Fig. 5) indicating that knockdown of AQP8 induced mitochondrial hyperpolarization.

### 3.4 Mitochondrial respiration in AQP8-knocked down cells

**Fig. 6:**
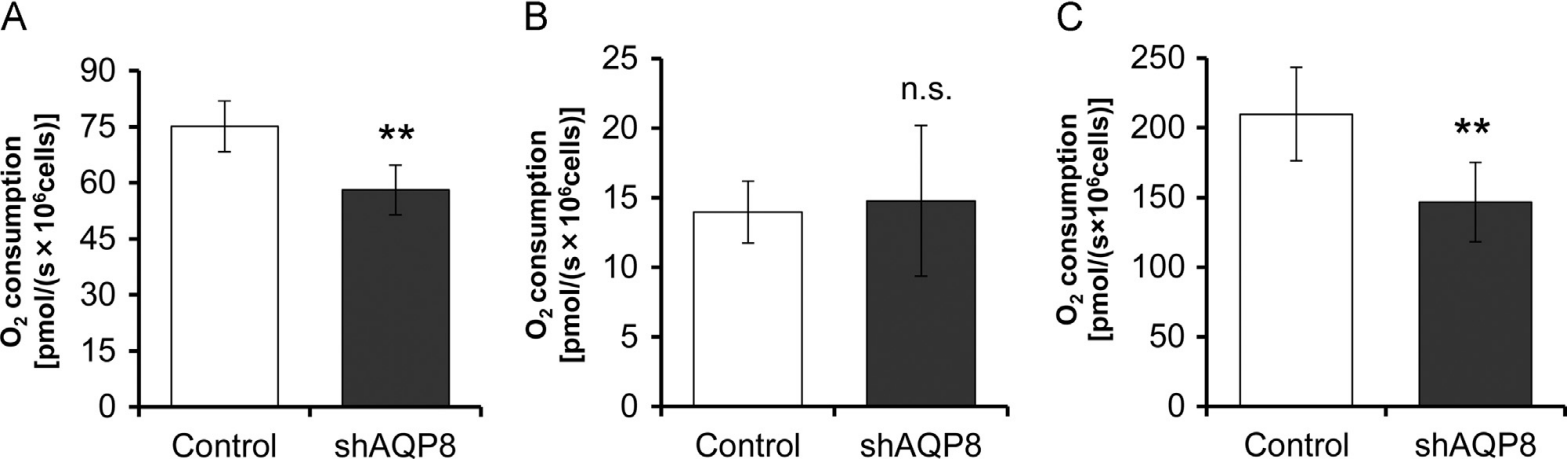
Mitochondrial respiration through different segments of the electron transport chain. Routine respiration (A), Leak respiration (B), which were obtained by inhibition of ATP synthesis with oligomycin A, and the maximum oxygen flux, which were obtained after adding mitochondrial uncoupler FCCP (C). Basal levels were obtained by inhibition of complex III with antimycin A. Data are expressed as the mean±S.D. of nine independent experiments. (**p<0.01; n.s., not significant).

Oxygen consumption in the shAQP8 cells and control cells was measured by the OROBOROS oxygraph-2k. The Routine level of oxygen consumption in the shAQP8 cells was significantly reduced; the Routine respiration rate of shAQP8 cells and control cells were 58.1±6.7 and 75.1±6.8 pmol/s×10^6^ cells, respectively (Fig. 6A). The Leak level obtained after inhibition of ATP synthesis with oligomycin A was not affected; the Leak oxygen consumption rates of shAQP8 cells and control cells were 14.8±5.4 and 14.0±2.2 pmol/s×10^6^ cells, respectively (Fig. 6B). The maximum uncoupled level was significantly reduced; the maximum oxygen consumption rates in the shAQP8 cells and control cells were 146.8±28.6 and 209.8±33.6 pmol/s×10^6^ cells, respectively (Fig. 6C). The calculated value, Routine-Leak (R-L), which gives the amount of respiration coupled to ADP phosphorylation was significantly reduced in the shAQP8 cells. These results indicated that the amount of mitochondrial respiration coupled to ADP phosphorylation was lowered by AQP8 knockdown.

### 3.5 Cytochrome c oxidase and ATP synthase activities in AQP8-knocked down cells

**Fig. 7:**
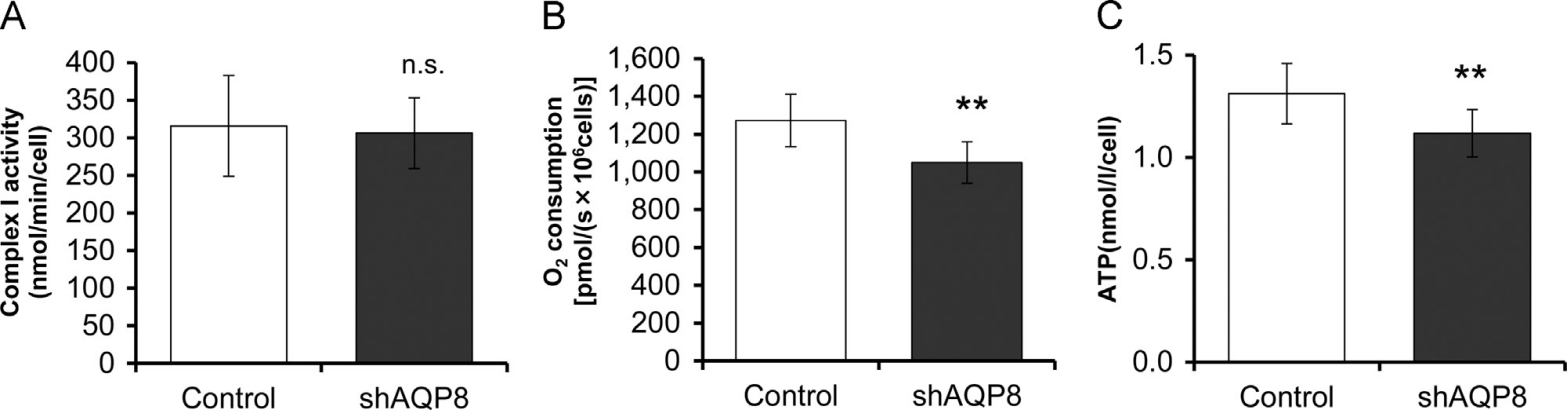
Activities of mitochondrial Complex I, IV and V. A: Complex I (NADH:ubiquinone reductase) activity assessed by diminution rate of NADH level (nmol/min/cell). B: Complex IV (cytochrome c oxidase) respiration measured with TMPD and ascorbate as substrates in the presence of antimycin A. C: Cellular ATP levels measured by luciferase assay. Data are expressed as the mean±S.D. of nine independent experiments. (**p<0.01; n.s., not significant).

**Fig. 8:**
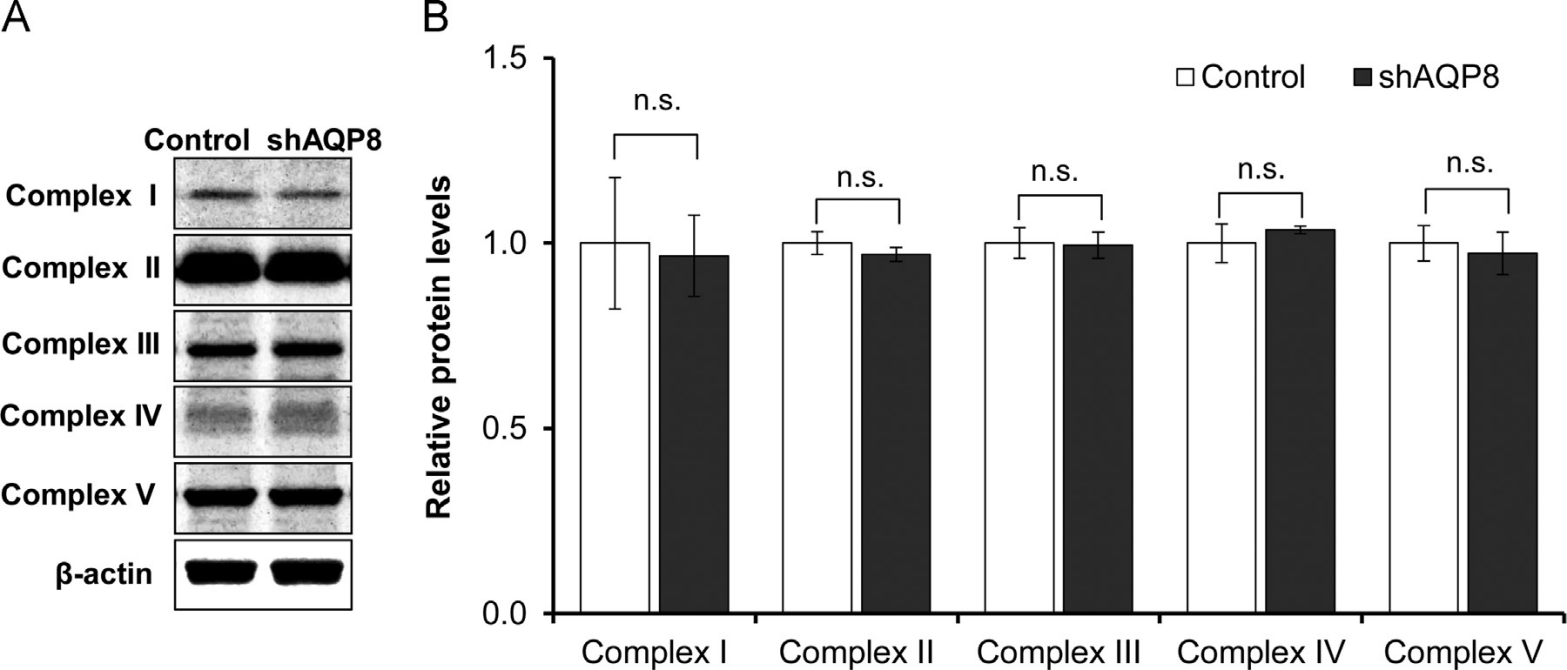
Protein levels of complex I-IV and the α-subunit of ATP synthase (complex V). Typical western blot images (A) and summarized expression levels (B) for oxidative phosphorylation complexes (Complex I–V) proteins. Each lane was loaded with 6 µg of protein. The protein levels normalized to those of β-actin were expressed as ratios against the controls. Data indicate the mean±S.D. of four independent experiments. (n.s., not significant).

**Fig. 9:**
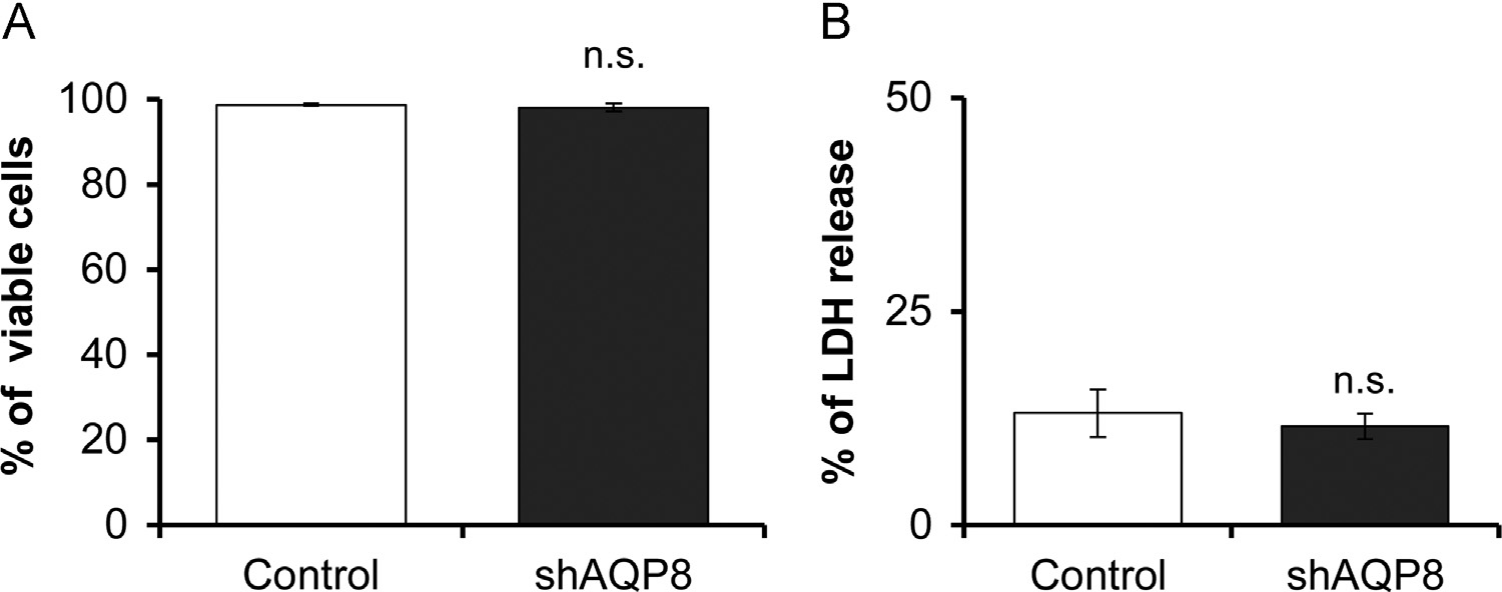
Cell viability studies. Cell viabilities in culture medium for 24 h was evaluated by Trypan blue assay (A) and in the medium without serum for 1.5 h was evaluated by LDH release assay (B). Data are expressed as the mean±S.D. of four independent experiments. (n.s., not significant).

We examined the effect of AQP8 knockdown on the activities of cytochrome c oxidase (complex IV) and ATP synthase (complex V), the two enzymes which produce large amounts of metabolic water. The activity of cytochrome c oxidase was assessed in the oxygen consumption experiments by adding *N*, *N*, *N*’, *N*’’-Tetramethyl-*p*-phenylenediamine dihydrochloride (TMPD) and ascorbate after adding the complex III inhibitor, antimycin A. The activity of ATP synthase was determined by measuring cellular ATP levels by luciferase assay. In the AQP8-knocked down cells, the activities of both of the two components involved in the production of metabolic water, were significantly lower than in the controls (Fig. 7B and C). On the other hand, the complex I activity was not affected by AQP8 knockdown (Fig. 7A). Concerning the protein expression level, none of the oxidative phosphorylation complexes I–V was affected by AQP8 knockdown (Fig. 8). Further, results from Trypanblue assay (Fig. 9A) and LDH assay (Fig. 9B) revealed that AQP8 knockdown had no effect on cell viability.

### 3.6 Mitochondrial morphology in AQP8-knocked down cells

**Fig. 10:**
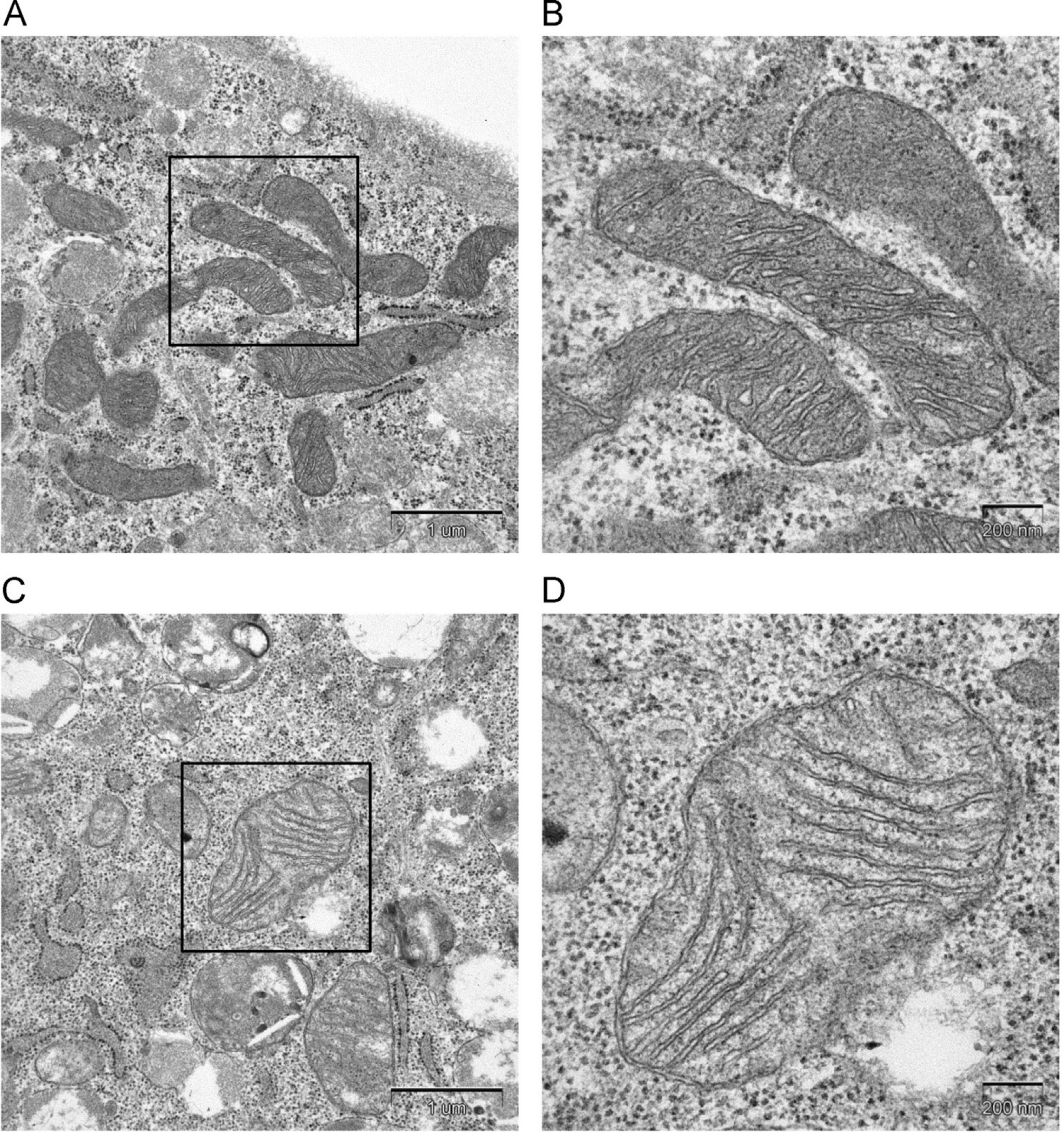
Electron micrographs of mitochondria. Typical transmission electron microscopy images of mitochondria in a control cell (A and B) and in an shAQP8 cell (C and D). The right images (B and D; scale bar, 200 nm) show higher magnification on the left (A and C; scale bar, 1 µm), respectively.

Mitochondrial morphology was observed by transmission electron microscopy of whole cells. It was noticed that the mitochondria were swollen and were more electron-lucent in the shAQP8 cells (Fig. 10C and D), compared with in control cells (Fig. 10A and B). The cristae in the mitochondria of shAQP8 cells, however, were still tightly packed. These results suggest that AQP8 knockdown causes mitochondrial swelling, consistent with a role of AQP8 in the regulation of mitochondrial water permeability.

## 4 Discussion

It has been reported that adipocytes express AQP3, 7, 9, 10 and 11 [24] on the plasma membrane. These AQPs are aquaglyceroporins, a subset of the AQP family, which has the permeability not only for water but also for glycerol, a substrate of lipogenesis and a product of lipolysis in adipocytes. It appears that the aquaglyceroporins in adipocytes are responsible for the flux of water and glycerol across the plasma membrane [25]. In this report, we demonstrated that adipose tissues and preadipocyte 3T3–L1 cells also express AQP8, which has permeability for water but not for glycerol. The majority of AQP8 in adipose tissues and 3T3–L1 cells was the unglycosylated type and was localized in the mitochondria. Based on these results, we considered that 3T3–L1 cells would provide a model system for studying the role of AQP8 in the mitochondria. We undertook knockdown of AQP8 in 3T3–L1 cells and established a clone, shAQP8, whose expression level was about one forth of the wild type.

**Fig. 11:**
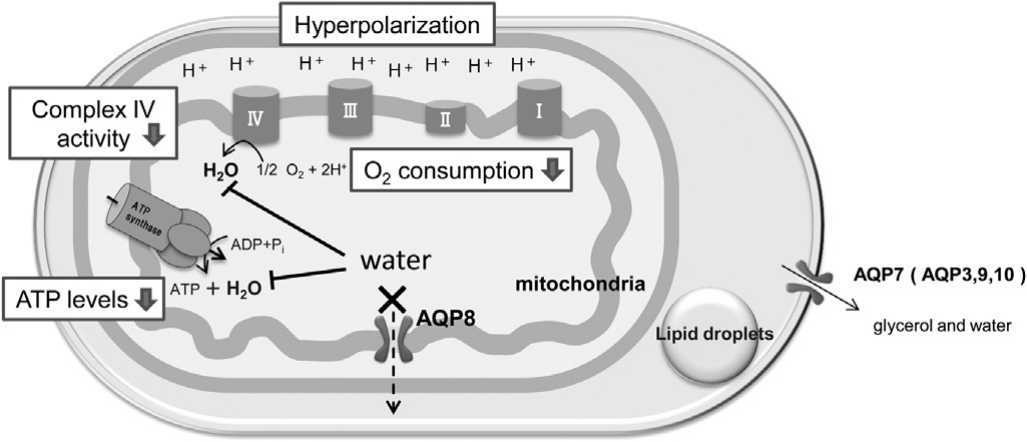
Predicted schema of mitochondrial dysfunction in AQP8-knocked down cells. In 3T3–L1 cells, AQP8 is localized in the mitochondrial membrane and is responsible for the efflux of water generated by oxidative phosphorylation. Its knockdown results in impaired mitochondrial water efflux and inhibits complex IV of the electron transfer system and ATP synthase, the steps in the ATP synthesizing pathway which yield water as a byproduct. The inhibition of complex IV results in reduced O_2_ consumption, and the inhibition of ATP synthase, a consumer of the proton gradient, causes mitochondrial hyperpolarization.

The AQP8-knocked down (shAQP8) cells we established appeared to have impaired mitochondrial function. It showed low routine level and low maximum capacity of respiration (Fig. 6A, C), while the protein levels of oxidative phosphorylation complexes, complex I–V, was not altered (Fig. 8). These results suggest that the rate of oxygen consumption per unit of the electron transport chain was decreased in the shAQP8 cells. The cellular ATP level in shAQP8 cells was significantly reduced to approximately 85% of that in controls, which had no apparent effect on the viability of cells (Fig. 9). The activities of two enzymes which produce water as a byproduct, cytochrome c oxidase and ATP synthase, but not complex I, were significantly reduced by AQP8 knockdown (Fig. 7). This was not accompanied by changes in the expression levels of these proteins (Fig. 8). These observations lead us to the hypothesis that the reduced AQP8 expression disrupts normal mitochondrial water flux, and lowers electron transport activity and ATP synthesis through inhibition of the water-generating processes (Fig. 11).

In shAQP8 cells, marked swelling of the mitochondria was observed by electron microscopy (Fig. 10C and D). This implies that AQP8 is indeed responsible for water excretion from the mitochondria in 3T3–L1 cells and that its knockdown affects mitochondrial function. To clarify the precise mechanisms of mitochondrial swelling, further studies including three-dimentional analysis of the dynamic changes in mitochondrial morphology, measurement of intramitochondrial osmolarity and the activities of mitochondrial transporters are needed. One of the factors which might affect mitochondrial volume regulation is the permeability transition pore (PTP), which opens in response to various stimuli such as increase in intracellular Ca^2+^ concentration and is accompanied by mitochondrial depolarization [26]. In case of the shAQP8 cells, the mitochondrial membrane potential was rather hyperpolarized, which suggests that the opening of PTP is not the major cause of decreased mitochondrial function.

The effect of AQP8 knockdown appears to be different among cell types. Transient knockdown of AQP8 in HepG2 cells was reported to result in mitochondrial depolarization and cell death, which was attributed to reduced H_2_O_2_ extrusion from the mitochondria [8]. In contrast, in shAQP8 cells, the mitochondrial membrane was hyperpolarized (Fig. 5), and the cells were viable and differentiation towards adipocytes could be induced. This might be related to difference in cellular localization and function of AQP8; in the hepatocytes, AQP8 was expressed both on the plasma membrane and in the mitochondria while, in 3T3–L1 cells, AQP8 was localized to the mitochondria.

The adipose tissue has not received much attention in mitochondria research so far. We report here, for the first time, that AQP8 is expressed in adipocytes. AQP8 was expressed in the mitochondria and its suppression resulted in reduced mitochondrial respiration. Cellular mitochondrial density and expression level of proteins related to mitochondrial respiration increases during adipocyte differentiation [27], [28] suggesting that mitochondrial respiration is essential for some adipocyte-specific function. In addition, the relationships between mitochondrial dysfunction and type 2 diabetes mellitus has been studied [29]. For example, inhibition of mitochondrial respiration in adipocytes by knocking down mitochondrial transcription factor A (TFAM) or application of several mitochondrial respiration inhibitors induced insulin resistance [30], [31] and impaired adipokine secretion [32]. In this connection, several important questions remain to be answered. Whether or not AQP8 is (i) co-regulated with the proteins in mitochondrial respiration, (ii) related to the specific function in adipocytes, and (iii) involved to pathological changes of adipocytes in type 2 diabetes. The shAQP8 cells established in the present study should provide a useful experimental system for the investigation of these questions.
